# Acquisition of antibodies to merozoite surface protein 3 among residents of Korogwe, north eastern Tanzania

**DOI:** 10.1186/1471-2334-10-55

**Published:** 2010-03-08

**Authors:** Method D Segeja, Bruno P Mmbando, Misago D Seth, John P Lusingu, Martha M Lemnge

**Affiliations:** 1 National Institute for Medical Research, Tanga Medical Research Centre, PO Box 5004, Tanga, Tanzania

## Abstract

**Background:**

A polymorphic malaria parasite antigen, merozoite surface protein 3 (MSP3), is among the blood stage malaria vaccine candidates. It is believed to induce immunity through cytophilic antibodies that disrupt the process of erythrocytes invasion by merozoites. This study aimed at assessing natural acquisition of antibodies to MSP3 in individuals living in an area with different malaria transmission intensity in preparation for malaria vaccine trials.

**Methods:**

The study was conducted in individuals aged 0-19 years from villages located in lowland, intermediate and highland strata in Korogwe district, northeastern Tanzania. Blood samples from 492 study participants were collected between May and June 2006 for malaria diagnosis and immunological investigations. Reactivity of MSP3 to different types of antibodies (immunoglobulin M, G and IgG subclass 1 and 3) were analysed by Enzyme Linked ImmunoSorbent Assay (ELISA).

**Results:**

Malaria parasite prevalence was higher in the lowland (50%) compared to the intermediate (23.1%) and highland (9.8%) strata. Immunogloblin G subclasses 1 and 3 (IgG1 & IgG3), total IgG and IgM were found to increase with increasing age. IgG3 levels were significantly higher than IgG1 (p < 0.001). Furthermore, *Plasmodium falciparum *infection was associated with higher IgG3 levels (p = 0.008). Adjusting by strata and age in individuals who had positive blood smears, both IgG and IgM were associated with parasite density, whereby IgG levels decreased by 0.227 (95%CI: 0.064 - 0.391; p = 0.007) while IgM levels decreased by 0.165 (95%CI: 0.044 - 0.286; p = 0.008).

**Conclusion:**

Individuals with higher levels of IgG3 might be partially protected from malaria infection. Higher levels of total IgG and IgM in highlands might be due to low exposure to malaria infection, recent infection or presence of cross-reactive antigens. Further studies of longitudinal nature are recommended. Data obtained from this study were used in selection of one village (Kwashemshi) for conducting MSP3 phase 1b malaria vaccine trial in Korogwe.

## Background

Malaria is one of the most serious public health problems in the world, affecting tropical developing countries and killing young children mainly [1]. Relationship between malaria morbidity and antibody levels to malaria antigens has been analyzed in several prospective longitudinal studies performed in different parts of Africa and Asia [2,3]. Adults develop potent but none sterile immunity against malaria in which individuals chronically harbor low grade parasitaemia and only occasionally suffer from mild clinical state known as premunition [4,5].

Currently, a number of malaria vaccines candidates are at different stages of clinical development. Promising results have been obtained with some of the vaccine candidate [6-8]. Among the blood stage candidate vaccines, merozoite surface protein 3 (MSP3) of *Plasmodium falciparum *offers good prospects for a potent vaccine whereby epidemiological and laboratory data suggest that, immune responses targeting this antigen is associated with protection [9]. It is believed that immunity induced by MSP3 is through cytophilic antibodies that disrupt the process of invasion of erythrocytes by merozoites [10]. Antibodies can exert their inhibitory function by preventing merozoite invasion into erythrocytes [11], by activating monocytes via cytophilic effective IgG1 and IgG3 isotypes [12] or by inhibiting cytoadherence of infected erythrocytes. Previous studies have demonstrated that immunoglobulin G (IgG) from individuals who are immune to malaria could passively transfer immunity to naïve infected recipients [11]. Immunoglobulin G cooperates with monocytes in a mechanism of antibody-dependent cellular inhibition of parasite growth (ADCI) *in vitro *[13]. Individuals protected against malaria produce mostly cytophilic antibodies (IgG1 or IgG3), whereas non-protected subjects produce mostly IgG2 and IgM [2,14]. Clinical trials of MSP3 vaccine in healthy, semi-immune adult males in Burkina Faso and children aged 12 to 24 months old in Tanzania showed it was safe and immunogenic [15,16].

The aim of this study was to employ a standardized ELISA assay to assess natural acquisition of antibodies to MSP3 in individuals living in an area with different malaria transmission intensity in preparation for malaria vaccine trials.

## Methods

### Study area and population

This study was conducted in Korogwe district, north-eastern Tanzania. The district is about 100 kilometers inland from Tanga. The population of Korogwe district in year 2002 (National census survey) was estimated to be 261,004, with a growth rate of 1.4% per annum. The area is topographically stratified into three strata namely; lowland, intermediate and highland. The strata are characterized by marked differences in malaria transmission profiles. The altitude of Korogwe district ranges from 300 - 1,200 meters above sea level (mASL). In these areas, malaria transmission decreases with increasing altitude [17,18] and is highest during and following the long rainy season, which usually extends from March through July. Low transmission is experienced during short rains of October - December. However, a recent study has shown non-significant differences in malaria prevalence between the two seasons [19].

In Korogwe district, the estimated average entomological inoculation rate (EIR) ranges in the past from 30-100 infective bites per person per year [17]. In lowland villages of Korogwe, malaria is perennial with peak seasons during and just after the rain [20]. *P. falciparum *is the predominant malaria species accounting for a little over 90% of all infections, the rest being *P. malariae *and *P. ovale *[21]. In Korogwe District Hospital, malaria is the leading cause of admission and deaths among underfive children [22].

Study villages were grouped into three strata namely; lowland, intermediate and highland based on the altitude and level of transmission. Mng'aza village was categorized as lowland stratum, Kwashemshi village as intermediate while Magundi, Kwamhanya and Vugiri villages were grouped into highland stratum. The study involved individuals aged 0-19 years. The details of the study villages are as explained elsewhere [19].

### Study design

This study was part of a longitudinal project being conducted in Korogwe. Samples used in this study were obtained from a cross sectional malaria survey conducted in May and June 2006 [19]. Plasma samples from 492 individuals (66 lowlands, 121 intermediate and 305 highlands) were analyzed by Enzyme Linked ImmunoSorbent Assays (ELISAs). A sample size of 89 samples from each stratum was estimated assuming a proportional mean difference of antibody levels to MSP3 between any two strata to be 30% and coefficient of variation of 90%; this variation has been also shown to vary with transmission elsewhere [23]. Thus, a minimum sample size of 270 was required from the three strata. For the purpose of assessing the effect of age on acquisition of antibodies, individuals were categorized into four age-groups 0-2, 3-4, 5-9 and 10-19 years. Over sampling was done in the highland area, because the villages were initially earmarked for MSP3 phase 1b vaccine trial [24].

### Ethical consideration

Ethical clearance was granted by the Medical Research Co-ordinating Committee of the National Institute for Medical Research and the Ministry of Health and Social Welfare, Tanzania. Prior to commencement of the study, sensitization meetings were held in each village to explain the objectives and methodology of the study as well as seeking for community consent. Informed consent was obtained in writing from all individual participants or parents/guardians in case of children during the survey as described elsewhere [19].

### Blood sample collection

Clinical examination was done in all 492 individuals at a central point in each village. Venous blood (about 3 ml) from each individual was drawn into vacutainer containing EDTA anticoagulant. Out of the 3 ml of blood collected, 8 μL was used for preparation of thick and thin blood smears for malaria parasite diagnosis, species identification and quantification. Also, 10 μL of blood of each individual was used for estimation of haemoglobin using Haemocue^® ^machine. The remaining blood was centrifuged at 2,000 revolutions per minute (rpm) to obtain plasma, packed red blood cells (RBCs) and buffy coat. Plasma was stored at -80°C and later analyzed for immunological assays.

### Determination of antibody responses to MSP3 by ELISA

Plasma samples were used to determine levels of IgM, total IgG and IgG subclasses (IgG1 & IgG3) responses to MSP3 malaria vaccine candidate by Enzyme Linked ImmunoSorbent Assay (ELISA) technique as described elsewhere http://www.amanet-trust.org. Briefly, wells of the microtiter plates (Nunc, Roskilde, Denmark) were coated with 100 μL at 0.5 μg/ml of a recombinant protein covering the nonpolymorphic carboxy-terminal region of the MSP3 protein (aa 212 - 380) [10]. The antigen was supplied as a stock solution of 2.4 mg/ml and had to be diluted so as to obtain a working concentration of 0.5 μg/ml. The plates were incubated overnight at 4°C, washed four times and blocked using (150 μL per well) 3% skimmed milk in phosphate buffered saline (PBS) -Tween 20 (Blocking Buffer) for 1 hour. Plasma samples (100 μL/well) at dilutions of 1:200 (for total IgG and IgM) and 1:50 (for IgG subclasses) were added in duplicate wells and incubated at room temperature (20 - 24°C) for 2 hours. Plates for IgG1 and IgG3 were then incubated for 1 hour with monoclonal mouse anti-human IgG1 and IgG3 (Skybio, Wyboston Bedfordshire, UK) as secondary antibodies at dilutions of 1:2000, and 1:5000 respectively. The plates were washed four times between aforementioned steps. Colour development was achieved either with horseradish peroxidase-conjugated goat anti-human total IgG and IgM (Caltag, Burlingame, CA) respectively, or horseradish peroxidase-conjugated goat anti-mouse IgG (Caltag, Burlingame, CA) for IgG subclasses followed by H_2_O_2 _with tetramethylbenzidine (TMB) substrate (Kem-En-Tec diagnostics, Kuldyssen, DK). Absorbance was read at 450 nm with reference at 650 nm. For each antibody isotype, a calibration (standard) curve was obtained from two-fold serially diluted purified myeloma proteins coated directly to duplicate wells of the first two columns of each plate. First well concentrations in ng/ml were: 100 (IgG & IgG3), 500 (IgG1) and 250 (IgM). Four pooled plasma from clinically immune adult Liberians and two Danish donors never exposed to malaria were used as positive and negative controls respectively. Antibody units were calculated using the Afro Immuno Assay (AIA) Network protocol of 2005 volume 1.01 excel based curve fitting program as described elsewhere [25].

### Data analysis

All data were entered and verified in Microsoft Access while statistical analysis was done using R version 2.8.0 and STATA software version 8 (stata Corp., College station, TX). All antibody levels were transformed to normality using the log transformation. Malaria parasite densities were converted from parasites per 200 white blood cells (WBCs) to parasite per microliter (μL) by multiplying by 40. A multiple regression analysis was used to assess variables associated with antibody levels. A p-value < 0.05 was considered significant.

## Results

### Malaria infection

Table 1 gives the baseline characteristics of individuals living in the three strata during May/June 2006 cross-sectional survey. Out of the 492 selected individuals, 305 (62%) were from highland stratum, 121 (24.6%) were from intermediate stratum and 66 (13.4%) were from lowland stratum. Overall, malaria parasites prevalence was 20.5% (101 out of 492). Distribution of *P. falciparum *parasite prevalence in the three strata were 50.0% (95%CI: 37.4 - 62.6) in the lowland stratum, 23.1% (95%CI: 16.0 - 31.7) in the intermediate stratum and 9.8% (95%CI: 6.7 - 13.7) in the highland stratum. Intermediate stratum had low malaria parasite prevalence but its geometric mean parasite density (GMD) among the positive cases was the highest among the three strata (424 rings/μL; 95%CI: 187 - 964). Bednet use was highest (91.4%) in the intermediate stratum. Spleen rate was higher in the lowland (30.8%) compared to the other two strata.

**Table 1:** Baseline characteristics of individuals sampled from lowland, intermediate and highland strata in Korogwe, Tanzania

Strata -*Village*	Altitude (mASL) (n = sample size)	*P. falciparum *prevalence % (95% CI)	*P. falciparum *GMD **(95% CI)**^**δ**^	Splenomegaly prevalence % (95% CI)	Bednet use %
Lowland-*Mng'aza*	334 (n = 66)	50.0 (37.4,62.6)	316 (173, 578)	30.8(20.0, 43.4)	24.6
Intermediate-*Kwashemshi*	409 (n = 121)	23.1 (16.0,31.7)	424(187, 964)	5.8(2.4, 11.6)	91.4
Highland-*Magundi*	*638 (n = 128)*	*14.1 (8.6,21.3)*	*237 (133,421)*	*10.9 (6.1,17.7)*	*17.19*
-*Kwamhanya*	*761(n = 86)*	*5.8 (1.9,13.0)*	*314 (63,1562)*	*4.7 ((1.3,11.2)*	*58.14*
-*Vugiri*	*941 (n = 91)*	*7.7 (3.1,15.2)*	*2020 (271,15042)*	*1.1(0.3,5.9)*	*46.15*
Average(Highland)	780 (n = 305)	9.8 (6.7, 13.7)	409 (218, 765)	5.0 (2.8, 8.1)	37.4

n = number sampled, mASL = meters above sea level, GMD = Geometric mean density, % = percentage, 95%CI = 95% confidence interval, ^δ^Based on individuals who had positive slides only

### Immunoglobulin G1 (IgG1 subclass)

**Figure 1:**
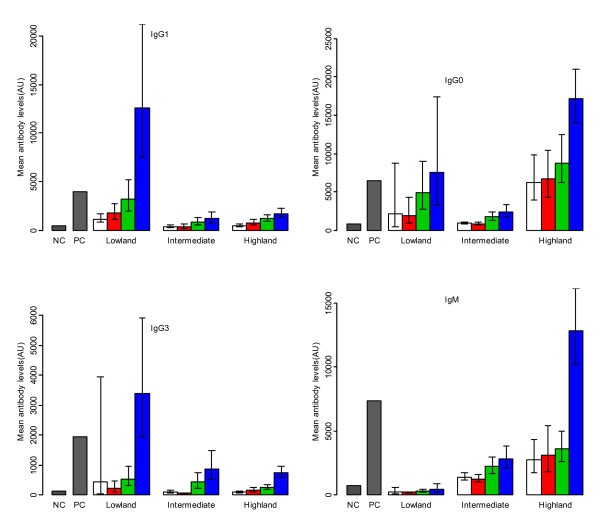
**Distribution of geometric mean levels of IgG1, IgG3, total IgG and IgM (AU) to MSP3 by age-groups and strata**. Children 0-2 years (open bars), 3-4 years (red bars) 5-9 years (green bars) and 10-19 years (blue bars) and line segments represent 95%CI. NC = Mean of negative controls and PC = mean of positive controls for respective antibodies against MSP3.

Individuals living in lowlands had higher IgG1 antibody levels compared to negative controls while individuals aged 10-19 years had higher mean levels than positive control (Figure 1). Results show that levels of IgG1 adjusted for other covariates were significantly lower in both intermediate by 1.653 (95%CI: 1.218 - 2.088; p < 0.001) and in highland strata by 1.229 (95%CI: 0.853 - 1.605; p < 0.001), when compared to the lowland stratum. Increase in mean haemoglobin concentration (Hb) by 1 g/dL was associated with decrease in IgG1 levels by 0.100 (95%CI: 0.008 - 0.192; p = 0.035). No statistically significant association was found between IgG1 levels and presence of *P. falciparum *parasites (Table 2).

**Table 2:** Linear regression estimates of parameters for variables associated with antibodies to MSP3 in Korogwe, Tanzania

Variable	IgG1	IgG3	IgM	IgG0
Intercept	8.363 (7.352, 9.374; p < 0.001)	5.324 (4.154,6.494; p < 0.001)	4.672 (3.651,5.693; p < 0.001)	6.906 (5.871,7.941; p < 0.001)
Intermediate stratum	-1.653 (-2.088,-1.218; p < .001)	-0.917 (-1.419,-0.415; p <0.001)	2.141 (1.702,2.580; p < 0.001)	-1.168 (-1.613-0.723; p < 0.001)
Highland stratum	-1.229 (-1.605,-0.853; p < 0.001)	-0.848 (-1.283,-0.413; p <0.001)	2.997 (2.617,3.377; p < 0.001)	0.742 (0.358, 1.126; p < 0.001)
Age (years)	0.120 (0.093, 0.147; p < 0.001)	0.190 (0.159,0.221; p < 0.001)	0.089 (0.062,0.116; p < 0.001)	0.084 (0.057, 0.111; p < 0.001)
Presence of *P. falciparum*	0.161 (-0.155,0.477; p = 0.319)	0.498 (0.131, 0.865; p = 0.008)	0.239 (-0.080,0.558; p = 0.143)	0.101 (-0.222, 0.424; p = 0.540)
Bednet use	0.036 (-0.238,0.310; p = 0.798)	0.133 (-0.185,0.451; p = 0.411)	-0.338 (-0.614,-0.06; p = 0.017)	0.094 (-0.186, 0.374; p = 0.510)
Hb (g/dl)	-0.100 (-0.192,-0.008; p = 0.035)	-0.039 (-0.147,0.069; p = 0.476)	0.031 (-0.063,0.125; p = 0.515)	0.071 (-0.023, 0.165; p = 0.140)

IgG1 = Immunoglobulin G subclass 1, IgG3 = Immunoglobulin G subclass 3, IgG0 = Total Immunoglobulin G, IgM = Immunoglobulin M, Hb = haemoglobin, < = less than, = equal

### Immunoglobulin G3 (IgG3 subclass)

Individuals living in lowlands had higher IgG3 antibody levels compared to negative controls while individuals aged 10-19 years had higher mean levels than positive controls (Figure 1). A pattern similar to that of IgG1 antibody levels was seen for IgG3, with intermediate and highland strata having significantly lower mean antibody levels compared to lowland stratum (Table 2).

The increase in IgG3 levels with age was 0.190 (95%CI: 0.159 - 0.221; p < 0.001) and was slightly higher than in IgG1. Individuals who had *P. falciparum *infection had significantly higher log mean IgG3 levels by 0.498 (95%CI: 0.131 - 0.865; p = 0.008).

### Immunoglobulin G (Total IgG)

Individuals living in highlands had higher total IgG antibody levels compared to negative controls while individuals aged 10-19 years had higher mean levels than positive controls (Figure 1). Mean levels of total IgG were higher in highland stratum compared to intermediate and lowland strata (Figure 1). Levels of total IgG in log scale as shown in table 2, were significantly lower in the intermediate stratum by 1.168 (95%CI: 0.723 - 1.613; p < 0.001) and higher in the highland stratum by 0.742 (95%CI: 0.358 - 1.126; p < 0.001) compared to the lowland stratum. Individuals who had *P. falciparum *infection had significantly high levels of total IgG by log 2.552 (95%CI: 1.045 - 4.059, p < 0.001). Adjusting by strata and age among individuals who had positive blood smears, IgG levels were associated with parasite density, whereby, the log mean levels of IgG decrease was 0.227 (95%CI: 0.064 - 0.391; p = 0.007).

### Immunoglobulin M (IgM)

Individuals living in highland stratum had higher IgM antibody levels compared to negative controls while individuals aged 10-19 years had higher mean levels than positive control (Figure 1). Mean levels of IgM increased with increasing altitude, the highest levels being seen in the highland stratum (Figure 1). Log mean difference in IgM levels in the highland stratum was 2.997 (95%CI: 2.617 - 3.377; p < 0.001) and that of intermediate stratum was 2.141 (95%CI: 1.702 - 2.580; p < 0.001) when compared to the lowland stratum (Table 2). IgM levels were also associated with bednet use, where in individuals who mentioned to use bednets, log mean levels were lower by 0.338 (0.062 - 0.614; p = 0.017). Adjusting by strata and age among individuals who had positive blood smears, IgM levels were associated with parasite density, whereby, the log mean levels of IgM decrease was 0.165 (95%CI: 0.044 - 0.286; p = 0.008). Individuals using bednets had significantly (p = 0.017) lower IgM levels (Table 2).

## Discussion

A number of malaria vaccine candidates are at different stages of development and some are undergoing clinical trials. It is believed that immunity induced by MSP3 is through cytophilic antibodies that disrupt the process of invasion of erythrocytes by merozoites [10]. The aim of this study was to employ a standardized ELISA assay to assess natural acquisition of antibodies to MSP3 in individuals living in an area with different malaria transmission intensity in preparation for malaria vaccine trials.

The burden of malaria as shown by parasite prevalence and spleen rate was highest in the lowland and lowest in the highland strata as reported in other studies [17,19,20]. High bednet use (91.4%) in the intermediate stratum might be the main factor for the lower than expected malaria prevalence and splenomegaly in this stratum. Lowland rural settings are normally characterized by high malaria transmission intensity as shown in our recent publication [19].

The increase in levels of total IgG, IgM, IgG1 and IgG3 antibodies with increasing age might reflect cumulative exposure to malaria parasites and possibly gradual maturation of the immune system over time as reported in other studies [26,27]. Both intermediate and highland strata had significantly lower mean IgG1 levels suggesting lower exposure to malaria as evidenced by the low malaria prevalence and splenomegaly [20].

The high levels of IgG1 and IgG3 in the lowland stratum, where malaria transmission is higher, suggests frequent exposure to malaria parasite as previously reported elsewhere [28,29]. The increase of IgG3 levels with age was slightly higher than that of IgG1 levels which might indicate that individuals with higher levels of IgG3 might be partially protected from malaria infection as reported in previous studies [11,30].

Lowest malaria parasite prevalence in highlands in association with high total IgG levels, especially in those aged 10-19 years, might indicate recent history of malaria transmission in the highlands where malaria infection is usually low [17,20,28] or could be due to the presence of cross-reactive antigens. The low total IgG levels in the intermediate stratum where bednet use was very high (91.4%) is as reported elsewhere [31,32]. Previous studies using human sera from individuals in malaria endemic populations have found evidence of association between total IgG levels to MSP3 with a reduced subsequent risk of clinical malaria [2,3].

The highest IgM levels in highland stratum in older individuals might suggest less exposure to malaria infection. It is known that non-protected individuals produce IgM antibodies mainly [2,14]. Furthermore, low IgM levels seen in the lowland stratum might be due to the continuous and intense malaria transmission. Notwithstanding this, individuals using bednets had significantly lower IgM levels.

## Conclusion

Individuals with higher levels of IgG3 might be partially protected from malaria infection. Higher levels of total IgG and IgM in highlands might be due to low exposure to malaria infection, recent infection or presence of cross-reactive antigens. Further studies of longitudinal nature are recommended. Data obtained from this study were used in selection of one village (Kwashemshi) for conducting MSP3 phase 1b malaria vaccine trial in Korogwe.

## List of abbreviations used

ml: millilitre; RBCs: red blood cells; IgG0: total immunoglobulin G; IgG1: immunoglobulin G subclass 1; IgG3: immunoglobulin G subclass 3; IgM: immunoglobulin M; EIR: entomological inoculation rate; ELISA: enzyme linked immunoSorbent assay; EDTA: ethylenediaminetetraacetic acid; rpm: revolutions per minute; μL: microlitre; °C: degrees Celsius; PBS: phosphate buffered saline; CA: Canada; UK: United Kingdom; DK: Denmark; H_2_O_2_: hydrogen peroxide; TMB: tetramethlylbenzidine; nm: nanometer; ng/ml: nanogram per millilitre; μg/ml: microgram per millilitre; aa: amino acid; AIA: Afro Immuno Assay; AIC: akaike information criteria; n: number; mASL: metres above sea level; Hb: haemoglobin; g/dL: grammes per decilitre; AU: arbitrary unit; WBCs: white blood cells; Hb: haemoglobin; temp: temperature; <: Less than; =: equal to; NC: negative controls; PC: positive controls.

## Competing interests

The authors declare that they have no competing interests.

## Authors' contributions

MDS assisted with proposal writing, study design, blood sample collection, laboratory analysis of blood samples, data interpretation and manuscript writing. BPM assisted with study design, data analysis, data interpretation and manuscript writing. MDS assisted with blood sample collection, laboratory analysis of samples and manuscript writing. JPL and MML assisted with proposal writing, study design, supervision of sample analysis, data interpretation and manuscript writing. All authors read and approved the final manuscript.

## Pre-publication history

The pre-publication history for this paper can be accessed here:

http://www.biomedcentral.com/1471-2334/10/55/prepub
